# Exclusive multipotency and preferential asymmetric divisions in post-embryonic neural stem cells of the fish retina

**DOI:** 10.1242/dev.109892

**Published:** 2014-09

**Authors:** Lázaro Centanin, Janina-J. Ander, Burkhard Hoeckendorf, Katharina Lust, Tanja Kellner, Isabel Kraemer, Cedric Urbany, Eva Hasel, William A. Harris, Benjamin D. Simons, Joachim Wittbrodt

**Affiliations:** 1 Centre for Organismal Studies (COS) Heidelberg, Im Neuenheimer Feld 230, Heidelberg 69120, Germany; 2 Department of Physiology, Development and Neuroscience, University of Cambridge, Downing Street, Cambridge CB2 3DY, UK; 3 Cavendish Laboratory, Department of Physics, JJ Thomson Avenue, University of Cambridge, Cambridge CB3 0HE, UK; 4 The Wellcome Trust/Cancer Research UK Gurdon Institute, University of Cambridge, Tennis Court Road, Cambridge CB2 1QN, UK

**Keywords:** Neural stem cells, Neural progenitor cells, Multipotency, Asymmetric division, Retina, Medaka

## Abstract

The potency of post-embryonic stem cells can only be addressed in the living organism, by labeling single cells after embryonic development and following their descendants. Recently, transplantation experiments involving permanently labeled cells revealed multipotent neural stem cells (NSCs) of embryonic origin in the medaka retina. To analyze whether NSC potency is affected by developmental progression, as reported for the mammalian brain, we developed an inducible toolkit for clonal labeling and non-invasive fate tracking. We used this toolkit to address post-embryonic stem cells in different tissues and to functionally differentiate transient progenitor cells from permanent, bona fide stem cells in the retina. Using temporally controlled clonal induction, we showed that post-embryonic retinal NSCs are exclusively multipotent and give rise to the complete spectrum of cell types in the neural retina. Intriguingly, and in contrast to any other vertebrate stem cell system described so far, long-term analysis of clones indicates a preferential mode of asymmetric cell division. Moreover, following the behavior of clones before and after external stimuli, such as injuries, shows that NSCs in the retina maintained the preference for asymmetric cell division during regenerative responses. We present a comprehensive analysis of individual post-embryonic NSCs in their physiological environment and establish the teleost retina as an ideal model for studying adult stem cell biology at single cell resolution.

## INTRODUCTION

Embryonic progenitor and stem cells generate new differentiated cells during the initial phases of development. Post-embryonic stem cells deal with a rather different issue, which is the addition of new cells to already functional organs. The switch from embryonic to post-embryonic stem cells is in general accompanied by loss of potency: while pluripotent cells can generate all cell types during early embryogenesis, adult organs and tissues are usually maintained by lineage-restricted post-embryonic stem cells (Blanpain and Fuchs, 2009; Van Keymeulen et al., 2011; Zhu et al., 2011).

Fish display a unique feature among vertebrates, which is their constant allometric growth beyond sexual maturity. All cell types are constantly added to every organ, making fish an ideal model to study post-embryonic stem cells and particularly how stem cell potency is affected during the transition from early embryo to juvenile and adult. Stem cell lineage analysis in fish has been largely performed by transplantation or by DNA injection, but both methods require the analysis of hundreds of clones to exclude putative technical artifacts (Centanin et al., 2011; Tu and Johnson, 2011; Wong and Rapaport, 2009). By contrast, genetic labeling of individual cells among a population (Livet et al., 2007) has proven to be an extremely useful, non-invasive tool for the analysis of embryonic and post-embryonic stem cells (Bonaguidi et al., 2011; Gupta and Poss, 2012; Rinkevich et al., 2011; Snippert et al., 2010; Loulier et al., 2014). Cre-mediated recombination was recently validated in fish (Gupta and Poss, 2012; Hans et al., 2009; Knopf et al., 2011; Mosimann et al., 2011; Nakamura et al., 2010; Singh et al., 2012), allowing long-term lineage of stem cells of embryonic origin (Pan et al., 2013).

The fish neural retina (NR) is ideally suited for comprehensively studying individual NSCs in a post-embryonic organ due to its stereotypic cell type distribution and spatio/temporal organization. It consists of seven main cell types distributed in three nuclear layers, and all these cell types are added continuously from the peripheral ciliary marginal zone (CMZ) (Amato et al., 2004; Johns, 1977; Reh and Levine, 1998; Centanin and Wittbrodt, 2014), which constitutes the niche of retinal stem cells (RSCs). In medaka, retinal NSCs were identified by the formation of arched continuous stripes (ArCoSs) (Centanin et al., 2011). In these experiments, permanently labeled blastula cells were transplanted into unlabeled blastula hosts. The formation of labeled ArCoSs containing all cell types of the neural retina demonstrated the existence of multipotent retinal NSCs of embryonic origin. However, early transplantation experiments do not allow the study of how changes in potency may occur after embryonic development due to the potential changes in niche and organ function. Particularly, the issue of whether cells in the post-embryonic neural retina are produced by multipotent NSCs or by the combined activity of several lineage-restricted – although still clonally related – NSCs could not be assessed with the described approach.

Here, we present a ‘living’ toolkit for individual post-embryonic stem cell research in medaka, and use it to address the localization and potency of post-embryonic NSCs with single cell resolution *in vivo* in their organismal context. Using inducible drivers for Cre recombinase, we demonstrate that post-embryonic NSCs always generate all cell types of the neural retina, including neurons and glia. Additionally, by labeling individual post-embryonic NSCs in the retina and following the resulting clone, we demonstrate a preferential asymmetric mode of cell division that is not changed after external challenges.

## RESULTS

### A medaka toolkit for life-long lineage analysis of individual stem cells

**Fig. 1.**
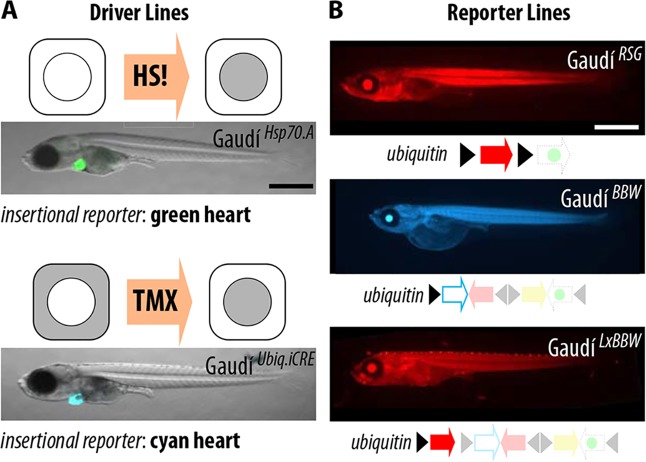
**A toolkit for post-embryonic clonal labeling in medaka.** (A,B) The toolkit is composed of two Cre-recombinase driver lines (A) and three LoxP reporter lines (B). (A) Cre transcription can be activated via heat shock in Gaudí^*HspCRE.A*^ (top, Cre represented in gray), which contains the integration reporter *cmcl2*:EGFP. Tamoxifen treatment will favor Cre nuclear translocation in Gaudí^*Ubiq.iCre*^ (bottom,Cre represented in gray), which contains the integration reporter *cmcl2*:ECFP. (B) Gaudí reporter lines express a default FP that is lost (DSRed in Gaudí^*RSG*^, top) or exchanged (Cerulean in Gaudí^*BBW2.1*^, middle; DS-Red in Gaudí^*LxBBW*^, bottom) upon Cre-mediated recombination. Scale bar: 1 mm.

To address individual post-embryonic stem cells, we developed a toolkit based on Brainbow constructs (Livet et al., 2007; Pan et al., 2013) that allows the induction of colorful mosaic medaka fish suitable for long-term lineage analysis (Fig. 1A,B). This living toolkit was named Gaudí after the Spanish architect famous for his colorful mosaics (supplementary material Fig. S1), and is composed of two alternative transgenic lines for inducible Cre expression and three fluorescent reporter lines to follow lineages (see Materials and Methods).

**Fig. 2.**
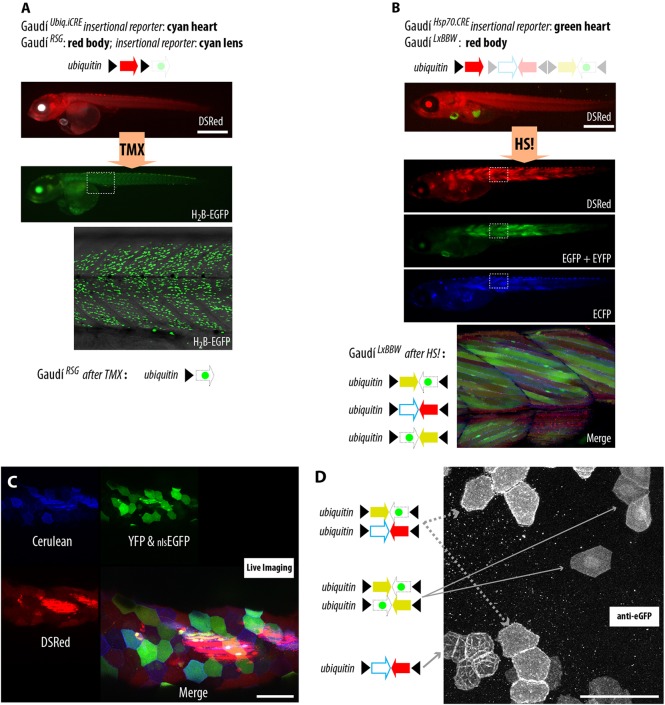
**Recombination can be determined in living and fixed samples.** (A) Tamoxifen induction leads to expression of H_2_B-EGFP in Gaudí^*RSG*^*,* Gaudí^*Ubiq.iCre*^ embryos. (B) A heat-shock treatment induces expression of Cerulean, YFP or H_2_B-EGFP in Gaudí^*LxBBW*^*,* Gaudí^*HspCre.A*^ embryos. Scale bar: 1 mm. (C) Live imaging of a recombined Gaudí^*LxBBW*^*,* Gaudí^*HspCre.A*^ fish allows identification of individual cells using native fluorescent proteins. Scale bar: 50 µm. (D) Immunofluorescence using a single anti-EGFP antibody allows detection of membrane-tagged Cerulean, cytoplasmic eYFP and nuclear eGFP in fixed samples of an adult cornea. Scale bar: 50 µm.

**Fig. 3.**
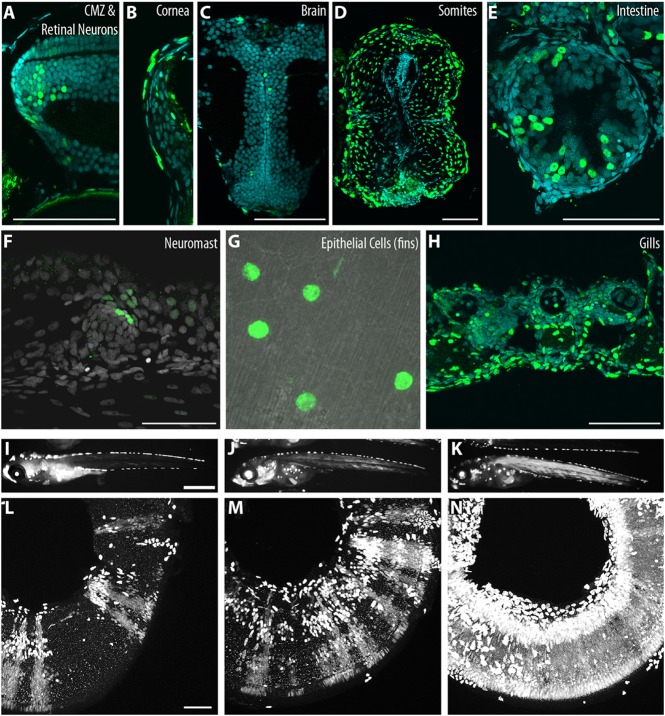
**Gaudí driver lines induce recombination in different tissues and have a large induction range.** (A) The Gaudí toolkit allows recombination in the CMZ and differentiated cells of the neural retina. (B-H) Recombination is also observed in different tissues such as cornea (B), brain (C), somites (D), intestine (E), neuromast (F), epithelia (G) and gills (H). (I-N) The number of recombined cells can be modulated from a few (I,L) to lot of cells (J,M) or almost the entire organ/tissue (K,N), modifying the intensity of the induction. Scale bars: 50 μm in A-H,L-N; 1 mm in I-K.

Gaudí^*HspCre.A*^ (Fig. 1A, top) contains a nuclear-tagged Cre recombinase, the expression of which is inducible upon heat-shock treatment until 10 days post-fertilization (*Hsp70*::_nls_CRE). Gaudí^*Ubiq.iCre*^ (Fig. 1A, bottom) contains a tamoxifen-inducible Cre recombinase under the control of a ubiquitous promoter (*ubiquitin*:^ERT2^Cre). For both lines, recombinase activity in most tissues is only detectable after induction (Figs 2 and 3; supplementary material Fig. S2).

Gaudí^*RSG*^ (Gaudí *Red-Switch-Green*; Fig. 1B, top) ubiquitously expresses a floxed DS-Red fluorescent protein, which prevents the expression of a nuclear-tagged EGFP. After Cre induction, the H_2_B-EGFP is evident in all recombined cells (Fig. 2A) and inherited by their progeny.

Gaudí^*BBW2.1*^ (Gaudí *Brainbow 2.1*; Fig. 1B, middle) offers additional fluorescent read-out for recombination. Upon Cre expression, the default membrane-tagged ECFP switches to one out of three alternative FPs: YFP, dTomato or _nls_EGFP (see supplementary material Fig. S2). This greatly expands the number of individual cells that can be followed within a tissue of interest.

Gaudí^*LxBBW*^ (Gaudí *floxed DSRed*,* Brainbow 2.1*; Fig. 1B, bottom) ubiquitously expresses a DS-Red FP, which is floxed-out upon Cre activation, allowing the expression of one out of four FPs: _nls_EGFP, YFP, _mem_ECFP and dTomato (Fig. 2B-D). Gaudí^*LxBBW*^ is the best option when fixation and immunostaining are required, as a single α-GFP antibody can be used to recognize three FP outputs based on their differential subcellular localization (Fig. 2C,D).

### The Gaudí toolkit permits labeling cells and lineage analysis of stem cells in most medaka tissues

To perform a proper lineage analysis, the reporter lines for recombination (LoxP-containing Gaudí lines, in this case) have to be expressed in every tissue and in every cell type of the organism, and the expression has to be maintained during the total chase or lineage time. Otherwise, the lineage will constitute only a fraction of the entire progeny, and the real potency of the stem cells studied will be underestimated. We detected the expression of the default or the alternative recombination read-out (fluorescent proteins expressed after Cre activation) in every embryonic and post-embryonic organ of the Gaudí reporter lines (Figs 1B, 2 and 3; supplementary material Fig. S3).

Both Gaudí^*HspCre.A*^ and Gaudí^*Ubiq.iCre*^ drive recombination in the CMZ (Fig. 3A), and in many other tissues such as the cornea, brain, somites, intestine, lateral line, epidermis and gills (Fig. 3B-H). One of the benefits of these inducible driver lines is that recombination levels can be adjusted by regulating the dose of the inducer (shift in temperature for Gaudí^*HspCre.A*^ and tamoxifen exposure for Gaudí^*Ubiq.iCre*^) (Bonaguidi et al., 2011). The experimental advantage of fish is their external development and the transparency of specific tissues even during larval stages, permitting screening under a fluorescence stereomicroscope. Using the Gaudí toolkit, the levels of recombination observed in the somites *in vivo* 2 days after induction (Fig. 3I-K) are a good proxy of the recombination that took place in the retina (Fig. 3L-N). We used this selection criterion for the experiments performed here, and rely on either sparse recombination (Bonaguidi et al., 2011) [in the case of the Gaudí^*RSG*^ line (see Material and Methods section)] or on just one of the possible read-outs in Gaudí^*BBW2.1*^ to reach clonality.

To validate the Gaudí toolkit as an appropriate method for lineage analysis *in vivo*, we compared it with alternative and already validated ways to label lineages in fish, namely DNA injection into the 2- to 4-cell stage embryos (Tu and Johnson, 2011) and transplantation at blastula stages (Centanin et al., 2011). Clones generated after DNA injection or transplantation experiments (supplementary material Fig. S4) reveal the lineage of cells labeled during early embryonic development. Induction of recombination at early embryonic stages using either Gaudí^*HspCre.A*^ or Gaudí^*Ubiq.iCre*^ in combination with Gaudí reporter lines resulted in the very same output (supplementary material Fig. S4A-C), as expected for a toolkit driving efficient labeling and allowing long-term lineage *in vivo*.

Definitive statements about stem cells involved in homeostasis (or homeostatic growth in the case of fish) need a late induction of clones, preferentially after functional development of the target organ is completed. We induced clones in Gaudí juvenile fish and grew them to adulthood (supplementary material Fig. S4). The resulting clones resembled the reported outputs for DNA injection and transplantation experiments in well-characterized tissues such as the fin (supplementary material Fig. S4D-G) (Tu and Johnson, 2011; Wong and Rapaport, 2009). The Gaudí kit confirmed the existence of post-embryonic, bona fide stem cells in a variety of organs throughout the entire fish (previous sections, and data not shown) and therefore constitutes a fundamental and versatile tool for the study of fish stem cells.

### The Gaudí toolkit reveals post-embryonic NSCs in the fish retina

**Fig. 4.**
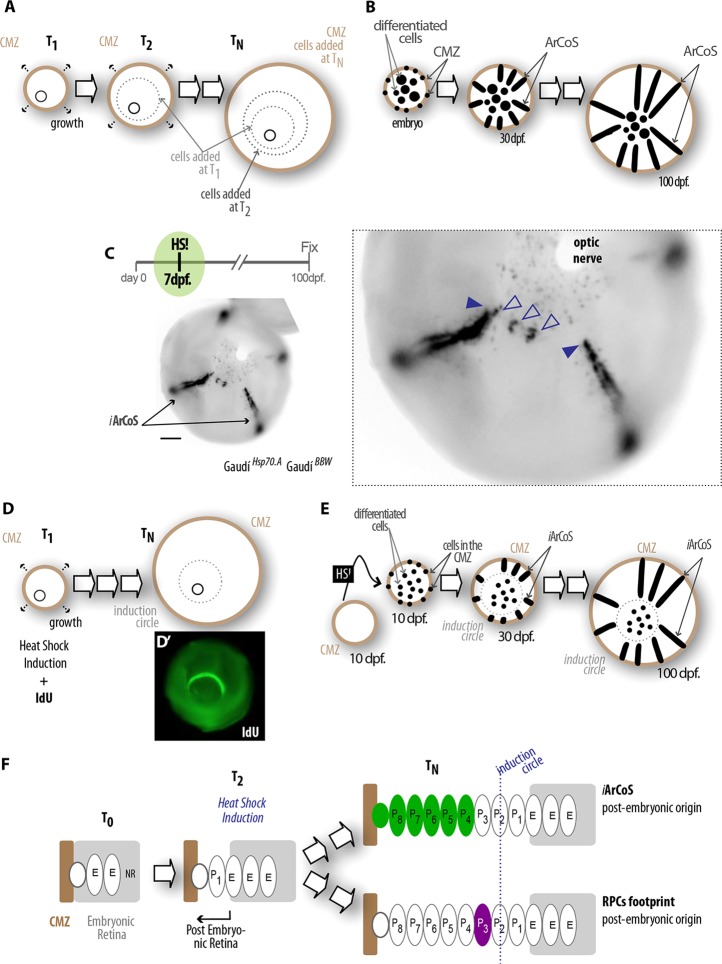
**Post-embryonic RSCs and RPCs**. (A) The medaka retina grows stereotypically by addition of cells in temporal concentric rings. (B) Transplantation of labeled blastula cells results in adult fish whose retinae contain clones of cells (ArCoSs) spanning from the embryonic to the adult retina. (C-F) Induction of Cre recombination at juvenile stage demonstrates post-embryonic retinal stem cells (RSCs) that generate induced ArCoS (*i*ArCoS) (C,E). IdU incorporated at the time of heat-shock induction helps visualizing the induction circle (D,E), which demarcates a time-ring at which recombination was induced months before (D’). A close look at the induction circle allows functional discrimination of RSCs versus retinal progenitor cells (RPCs). Although RSCs generate *i*ArCoS (C, filled arrowheads), transient RPCs generate smaller clones (C, empty arrowheads) that map closer to the induction circle than the origin of *i*ArCoSs (scheme in F), reflecting a more-central location in the ciliary marginal zone (CMZ). (E) Neurons produced during embryogenesis. P_1_, P_2_, P_N_: groups of neurons generated at different post-embryonic stages. Scale bar in C: 50 μm.

Neurons are added to the neural retina (NR) of fish in an extremely stereotyped manner and stay in that same place for the rest of the fish's life, with no mixing among neurons born at different time points (Johns, 1977). This precise spatio/temporal arrangement is referred to as a tree-like growth in concentric rings (Johns, 1977), where the peripheral addition of new neurons results in a central circle that contains the oldest, embryonic retinal cells and peripheral rings that are composed of newly differentiated cells (Fig. 4A). This form of growth was shown in several different fish species, such as goldfish, zebrafish and medaka (Johns, 1977; Allison et al., 2010; Centanin et al., 2011). In medaka, retinal stem cells (RSCs) of embryonic origin form ArCoSs, which are clonal stripes that extend perpendicular to the temporal rings, from the embryonic to the adult retina (Fig. 4B) (Centanin et al., 2011). Therefore, ArCoS formation is the way of defining stemness for a retinal cell in fish. In medaka, ArCoSs are composed of all seven main neuro-retinal cell types (Centanin et al., 2011).

We decided to use the Gaudí kit to characterize RSCs functionally in a mature retina. After inducing stochastic recombination of Gaudí reporter lines in juvenile fish, those showing recombination in the retina were selected and grown for 3 months. These retinae showed induced ArCoSs (*i*ArCoSs) (*n*=15/23 retinae, 65%) spanning from the juvenile to the adult retina (*n*=86 *i*ArCoSs distributed in 15 retinae, averaging 5.7 *i*ArCoSs per retinae) (Fig. 4C), highlighting post-embryonic RSCs. In contrast to embryonic ArCoSs, which extend out of the embryonic retina, *i*ArCoSs start at more peripheral positions, revealing their post-embryonic origin (Fig. 4C-E). This confirms that the NR contains genuine post-embryonic stem cells that sustain its growth during the entire life of the fish.

### Post-embryonic retinal NSCs and progenitor cells are located in distinct domains of the CMZ

In addition to the permanent RSCs, several studies of the adult teleost CMZ postulate the existence of transient retinal progenitor cells (RPCs) (Johns, 1977; Reh and Levine, 1998), as described in other stem cell niches (Mizutani et al., 2007; Rothenaigner et al., 2011). Notably, it has been especially challenging to functionally differentiate progenitor cells from genuine stem cells *in vivo*. We took advantage of the temporal arrangement of the retina to examine the site corresponding to the induction time (juvenile retina) (Fig. 4C-F), which can be easily demarcated by performing a short pulse of IdU when Cre-recombinase is induced (Fig. 4D, induction circle). Analysis of adult retinae from Gaudí^*HspCre.A*^ Gaudí^*BBW2.1*^ induced at juvenile stages revealed two different features of proliferating retinal cells. Besides *i*ArCoSs spanning from the induction circle to the peripheral retina (filled arrowheads, Fig. 4C), we identified short clones terminating soon after their birth time (45 short clones, *n*=10 retinae) (open arrowheads, Fig. 4C). These represent clonal footprints of progenitor cells that, due to their transient nature, only give rise to limited progeny. Strikingly, there is a stereotypic spatio/temporal gap between the footprint of progenitor cells and the first cells in an *i*ArCoS (*n*=17 RPCs footprints and 15 ArCoSs in five retinae). Given the tight spatiotemporal correlation of cell addition during fish retinal growth, this gap indicates that the ‘more central’ footprints of RPCs were incorporated earlier and the ‘more peripheral’ ArCoSs from RSCs started later (Fig. 4C,F; supplementary material Fig. S5, Movies 1 and 2). This in turn reflects the initial positions of RPCs and RSCs in the CMZ at the time of induction. Although RPCs are located more centrally and therefore their progeny exit the CMZ earlier, RSCs reside in an adjacent but more peripheral circle and it takes longer for their progeny to pass through the RPC domain before they are eventually integrated into the differentiated retina (supplementary material Fig. S5).

### RSCs and RPCs have different temporal requirements

**Fig. 5.**
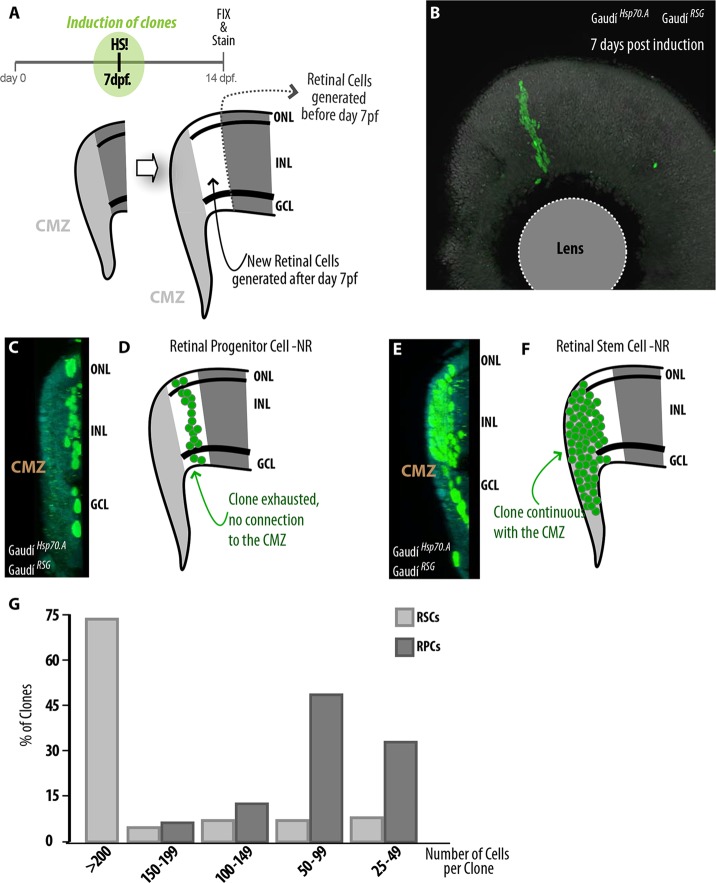
**Functional differences between post-embryonic RSCs and RPCs.** (A) The stereotyped addition of cells to the NR allows analyzing temporal aspects of post-embryonic neurogenesis. (B) Moderate induction of Gaudí fish results in sparse labeling of stem and progenitor cells forming isolated retinal clones. (C,D) 3D reconstruction of a clone generated by a RPC. The clone has detached from the CMZ and all labeled cells are already differentiated. (E,F) 3D reconstruction of a clone arising from a RSC. The clone is continuous with the CMZ, and a fraction of the older, i.e. more central, cells is already incorporated in the layered retina. (G) Distribution of number of cells per clone in RSCs and RPCs, 7 dpi. Most RSCs form big clones containing more than 200 cells, and most RPCs form clones of fewer than 100 differentiated cells.

As the spatio/temporal organization of the retina allows us to address RPCs separately from RSCs, we investigated for how long a RPC produces progeny before it is exhausted. We induced a few individual clones per retina and found that at 7 days post-induction (Fig. 5A,B) the progeny of RPCs was already detached from the CMZ (Fig. 5C,D; supplementary material Movie 1), indicating the exhaustion of proliferative cells in the clone. Complementarily, the progeny of labeled RSCs took 7 days to fill up the CMZ and to start contributing differentiated cells to the layered retina (Fig. 5E,F; supplementary material Movie 2). Therefore, lineage analysis of single cells in the context of the stereotypic growth occurring in the fish retina makes it possible to define the initiation and exhaustion of the proliferative capacities of RPCs and RSCs in their natural niche.

### Both RSCs and RPCs are multipotent, and each individual RSC generates all cell types in the NR

To address the potency and proliferative capacities of RSCs and RPCs, we analyzed the cell number and cellular composition of *i*ArCoSs and footprints (containing more than 20 cells), respectively. We induced sparse recombination in Gaudí^*RSG*^ and performed quantifications in the entire retina at 7 dpi. RSCs preferentially generated clones containing more than 200 cells (73.4%, *n*=214) (Fig. 5G), and in all cases these cells filled a complete retinal column distributed in all three nuclear layers. Conversely, most clones generated by RPCs (the footprints mentioned above) ranged from 25 to 99 cells (81.3%, *n*=64) (Fig. 5G), and some expanded to 170 differentiated retinal cells (6.3%, *n*=64). All RPC clones produced more than two cell types, ruling out the existence of dedicated, cell type-specific RPCs in the mature retina. This extends reports on RPC potential performed during fish retinogenesis (He et al., 2012) and the early frog CMZ (Wetts et al., 1989; Wong and Rapaport, 2009).

**Fig. 6.**
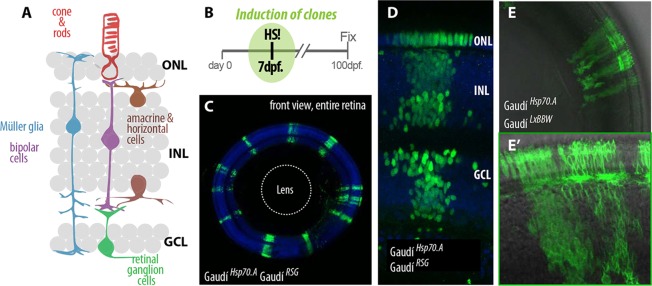
**Exclusive multipotency among post-embryonic RSCs.** (A) The stereotypic distribution of cell types in the differentiated retina facilitates the analysis of major retinal cell types. (B) Juvenile Gaudí fish are induced by Cre-mediated recombination and grown until adulthood. (C-E) Individual NSCs in the retina are multipotent. Every clone spans through the three retinal nuclear layers (C) and contains all mayor retinal cell types (D). Gaudí^*LXBBW*^ allows unambiguous assignment of *i*ArCoSs and demonstrates multipotency in adjacent RSCs (E, and detail in E′).

To address the full potency of retinal stem cells in the mature CMZ, we analyzed the cellular composition of *i*ArCoSs 2 months post-induction. The Gaudí toolkit in combination with the stereotyped organization of cell types in the NR allows us to study the potency of hundreds of individual RSCs (Fig. 6A-C). Lineage analyses indicated that every single NSC analyzed in the retina is multipotent (*n*>300) with progeny distributed over the three nuclear layers (Fig. 6D), as previously seen for shorter lineage times (Fig. 5E,F; supplementary material Movie 3). Even neighbor clones maintained by adjacent RSCs labelled by different outputs of the Gaudí^LxBBW^ were composed of cells that fill the entire retinal layers (Fig. 6E,E′). To confirm that RSCs exclusively generate *i*ArCoSs that contain every main cell type of the neural retina, we used specific antibodies against cell types in the inner nuclear layer. We found that all *i*ArCoSs analyzed were positive for the antibodies tested (*n*=97 iArCoSs; 63/63 PKCa^+^, bipolar cells; 77/77 GS^+^, Müller glia cells; 18/18 HuC^+^, horizontal and amacrine cells; 20/20 parvalbumin^+^, amacrine cells; 9/9 triple positives for GS, PKCa and HuC; data not shown). This demonstrates that each *i*ArCoS-forming RSC self-renews and always gives rise to the entire complement of neural retinal main cell types.

### Preferential asymmetric cell divisions of NSCs in the medaka retina

**Fig. 7.**
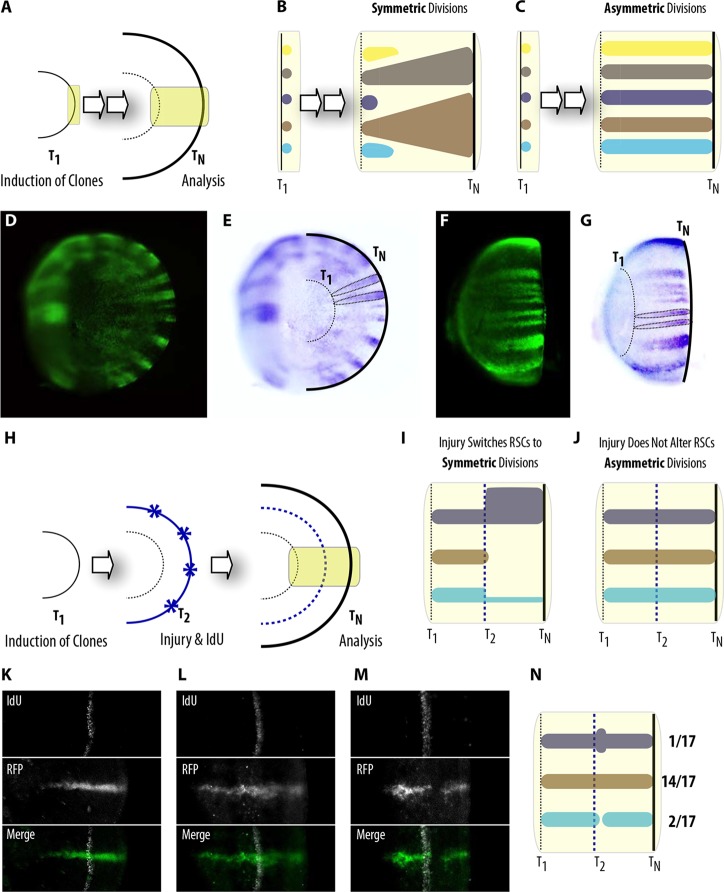
**RSCs undergo asymmetrical cell divisions during homeostatic growth and regeneration.** (A-C) Prediction of ArCoSs shape during growth of the retina (A), assuming symmetric divisions and neutral drift (B) or preferential asymmetric divisions (C) among RSCs. (D-G) All ArCoSs observed fit the asymmetric preference, even in old fish of 18 months of age. Inner (D) and outer (F) views of *i*ArCoS-containing retinae, and diagrams (E,G) indicating induction circle at T_1_ and final age at T_N_. (H-N) RSCs do not change their behavior during regeneration responses. (H) Scheme of the experimental timeline. (I) If RSCs change to symmetric divisions upon injury, *i*ArCoSs should either expand or reduce their width from the induction circle onwards. (J) A fixed choice for asymmetric divisions would result in constant width before and after the induction ring. Most of the *i*ArCoSs analyzed do not change their shape during regeneration (K,N). In some cases, there is a transient expansion (L,N) or reduction (M,N) in the width of the ArCoSs, indicating a response mediated by RPCs.

Recently, a number of reports analyzing diverse vertebrate post-embryonic stem cells at clonal resolution (Doupé et al., 2012; Klein et al., 2010; Mascré et al., 2012; Snippert et al., 2010) shifted the traditional view on stem cell behavior *in vivo* (Simons and Clevers, 2011). These studies sustain a model of homeostatic turnover in which equally potent stem cells undergo a continuous process of stochastic loss and replacement so that their overall population is maintained. Through this process of neutral competition, stem cells marked in a pulse-labeling assay become increasingly invested in an ever-diminishing population of surviving clones (see the expected progression of RSCs clones according to this model in Fig. 7A,B). Intriguingly, retinal NSCs in fish give rise to *i*ArCoS that are not lost nor do they displace other clones over time (Fig. 7C-G; see also Fig. 6C). Instead, *i*ArCoSs are stable throughout the life of the animal (98% of *i*ArCoSs generated in juvenile fish continue into the adult CMZ, *n*>200). These results suggest that retinal NSCs are maintained through a predominantly asymmetric mode of cell division.

This preferential mode of asymmetric divisions is maintained by RSCs of the NR throughout life. If there was a shift to symmetric divisions at some point during late adulthood, we would expect to observe a more stochastic scenario (Fig. 7A,B), i.e. the termination of some *i*ArCoSs and the widening of others. Even in the oldest fish analyzed (18 months after transplantation or induced recombination), ArCoSs and *i*ArCoSs maintained their continuous, consistently wide shape throughout life (*n*>60 ArCoSs, *n*>50 *i*ArCoSs).

### RSCs keep their division mode after injuries in the transient amplifying domain

Stem cell decisions taking place during homeostatic conditions can be altered when challenged by injuries, a feature widely reported for other post-embryonic stem cells (Blanpain and Fuchs, 2009; Van Keymeulen et al., 2011). To investigate whether the preference for asymmetric divisions is maintained in RSCs during regeneration, we used the highly ordered fashion in which cells are added to the fish retina to compare the width of a growing *i*ArCoS before and after injury. Gaudí^*Hsp*.Cre.A^, Gaudí^*BBW2.1*^ fish were induced for recombination at late embryonic stages, selected for ArCoSs formation and grown for 3 weeks. We then produced several small injuries at the boundary between the CMZ and the differentiated retina, treated the fish with IdU overnight and grew them for three additional weeks (scheme in Fig. 7H). The IdU pulse labeled the retinal neurons generated at the time of the injury all around the retina, and constitutes a temporal ring that separates the pre-injured from the post-injured retina – hereafter called injury ring (scheme in Fig. 7H).

Comparing the width of an *i*ArCoS before and after the injury allows us to assess whether or not RSCs changed their division preference. *i*ArCoSs that are terminated or whose width is increased soon after the ‘injury ring’ indicate a change from asymmetric to symmetric divisions (Fig. 7I). *i*ArCoS that maintain their width before and after the ‘injury ring’ point to a fixed mode of asymmetric division (Fig. 7J). In 14 out of 17 cases analyzed (*n*=8 retinae), we found that the width of the *i*ArCoSs did not change over the IdU ring (Fig. 7K), indicating that the preference for asymmetric cell division among RSCs was not affected by external stimuli such as injury.

We observed, however, three cases in which the injury triggered transient changes in the width of the *i*ArCoSs. Around the ‘injury ring’, 1/17 *i*ArCoSs expanded (Fig. 7L) and 2/17 *i*ArCoSs reduced (Fig. 7M) the region they previously covered, to fill in the injury with new neurons. This expansion/reduction was transient – as progenitor cells are – and the width of the *i*ArCoSs returned to its pre-injury dimension (Fig. 7L-N). Overall, our results indicate a fixed decision among RSCs concerning their preferred mode of cell division. We hypothesize that the plasticity of the proliferative capacity introduced by RPCs allows a transient response to compensate for injuries.

### Increase in RSC number during growth indicates minor symmetric cell divisions

**Fig. 8.**
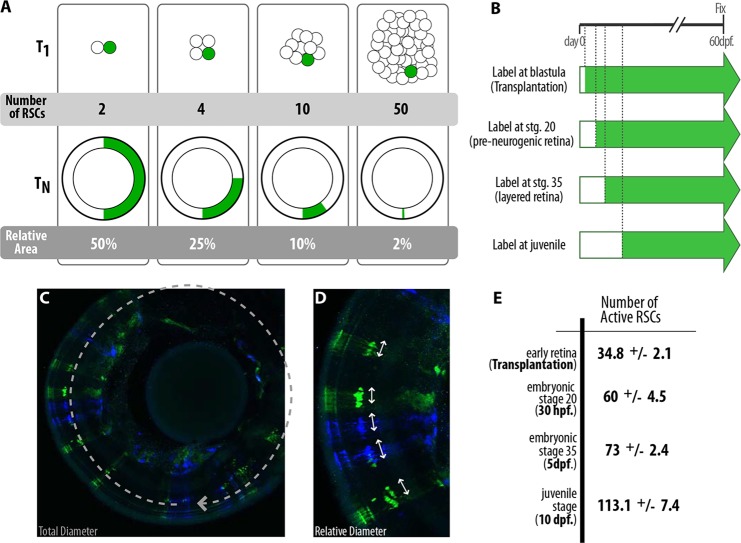
**RSCs also follow minor symmetrical cell divisions to increase stem cell number during homeostatic growth.** (A) Based on the relative clone size, it is possible to infer the number of active stem cells in an organ. (B-E) RSCs increase in number as the fish grows. Inducing recombination in Gaudí fish at different stages (B) results in *i*ArCoSs that differ in the relative occupancy of the retinal diameter (C, detail in D). The older the stage during which recombination was induced, the smaller the fraction of the retina occupied by each *i*ArCoSs and, therefore, the higher the number of active RSCs at the induction time (E).

We have previously shown that the medaka retina grows during embryonic and post-embryonic life. As the eyes increase in size, the CMZ also grows, hosting progressively more cells as growth proceeds. To investigate whether the number of active RSCs increases over time, we performed transplantation experiments at blastula stages, as well as Gaudí inductions at later time points. The fraction of the neural retina labeled by each clone (width of the clone relative to the circumference of the retina) constitutes a good proxy for the number of active stem cells at the time at which labeling was induced, provided that RSCs are all equally active (Fox et al., 2008) (see schemes in Fig. 8A,C,D). This method was successfully used to estimate the total number of stem cells in other systems (Margolis and Spradling, 1995; Ohlstein and Spradling, 2006).

Transplantation experiments at blastula stages revealed the presence of about 35 active RSCs in the neural retina at its very initial stage. We analyzed the relative occupancy of 106 ArCoSs in 2-month-old adult fish, and estimated 34.8±2.1 active RSCs present at the earliest retinogenic stage (Fig. 8B,E). The analysis of Gaudí fish induced at a pre-neurogenic retinal stage revealed the presence of 60±4.5 active RSCs. Induction of recombination in Gaudí fish induced at 5 dpf, in which most of the retina is already layered and the CMZ is evident, revealed that the number of active RSCs had already increased to 73±2.4 (*n*=89 ArCoSs) (Fig. 8B,E). Moreover, when Gaudí fish were induced at 10 dpf, the number of active RSCs had increased to 113.1±7.4 (Fig. 8B-E). Our data demonstrate that RSCs do indeed go through symmetric divisions to expand the number of active stem cells during eye growth.

## DISCUSSION

In this study, we developed a genetic toolkit that allows single cell labeling and long-term lineage analysis in medaka to gain insight into the functional properties of retinal stem cells (RSCs) *in vivo*. We generated and used the Gaudí toolkit to reveal the occurrence of RSCs in an already functional eye, and to demonstrate that individual RSCs generate the entire repertoire of cell types in the neural retina. We show that RSCs preferentially (but not exclusively) divide in an asymmetric way that is reflected in the life-long clone continuity. RSCs also divide (to a minor extent) in a symmetric way, which increases the stem cell number and sustains the equatorial expansion of the growing retina. Our results indicate two stereotypic behaviors among RSCs in medaka: (1) there is no variation in potency among individual stem cells in the fish neural retina; and (2) the preference for asymmetric divisions is not altered by age or during tissue repair.

### Homogenous potential of RSCs in the neural retina

When assessed by single cell labeling methods, progenitor cells have proved to be extremely heterogeneous regarding the number and cell types produced – irrespective of the system in which these were analyzed (Brock et al., 2009; Costa et al., 2011; Graf and Stadtfeld, 2008; Mascré et al., 2012; Rothenaigner et al., 2011). Several studies assessed the potency of individual RPCs in the early retina of different vertebrates (Fekete et al., 1994; Gomes et al., 2011; He et al., 2012; Holt et al., 1988; Price et al., 1987; Wong and Rapaport, 2009; Wetts and Fraser, 1988), and they all reported a scenario in which the number and cell types generated are highly variable among RPCs.

The situation we report for the homeostatic growth of the neural retina is far more uniform. We focused on RPCs with the potential of going through at least six more divisions (i.e. generating clones that contain more than 25 cells) and observed that all of them were multipotent. Furthermore, most of the RPCs analyzed were able to generate all seven main cell types present in the neural retina. Although our results neither overrule stochasticity during differentiation nor exclude the existence of dedicated RPCs, they clearly indicate that such a behavior would be restricted to the last cell divisions before cells acquire their post-mitotic identity. Our data indicate that a few divisions higher in the lineage, cells of different retinal cell types share a common progenitor cell.

Our short-lineage experiments revealed transient clones containing up to 170 cells. These clones could originate from either RPCs or RSCs that are displaced from the CMZ niche, as shown for stem cells in other systems (Snippert et al., 2010). The fact that these big clones are consistently terminated within a few days indicates a limitation in their proliferative potential. They could still be seen as originating from identical cells and resulting in a different output due to cell competition. However, we reproducibly observed that the terminated clones were closer to the center of the retina than the central edges of ArCoSs, arguing that these terminated clones arise from cells that were originally sited at a more central location in the expanding retina (supplementary material Fig. S5). Therefore, we argue that these cells with limited proliferative potential map to a defined more-central domain within the CMZ, the transit amplifying zone, and therefore represent RPCs. Our data show that the proliferative capacities of stem-cell-derived RPCs cover a wide range, and go beyond the mitotic capacities reported for early RPC during initial retinal neurogenesis in zebrafish and *Xenopus* (He et al., 2012; Holt et al., 1988; Wong and Rapaport, 2009; Wetts and Fraser, 1988).

This multipotent behavior of RPCs is highly reminiscent of that of stem cells in the medaka neural retina. In contrast to individual NSCs in rodents, which generate variable multi-lineage offspring (Bonaguidi et al., 2011; Encinas et al., 2011), we observe fully penetrant multipotency at the level of individual NSCs. This is massively expanding the present view on the potential of a single post-embryonic neural stem cell. Each *i*ArCoS-forming RSC self-renews and always gives rise to a continuous column that contains the entire complement of neural retinal cell types. Our results indicate no variability (and therefore no stochastic decisions) among RSCs regarding the cell types they generate in the neural retina.

The mandatory clonality of retinal columns raises the question of its functional implications. Remarkably, two publications have recently reported that columns of neurons in the mammalian cortex have a clonal origin that favors interconnection between siblings, establishing a link between clonal generation and coordinated function (Li et al., 2012; Yu et al., 2012). It will be exciting to address whether the clonal relation within neural *i*ArCoSs from photoreceptors to ganglion cells has similar functional implications, as observed in the mammalian cortex.

### Symmetric and asymmetric divisions among RSCs *in vivo*

The availability of genetic tools to label and lineage trace single cells has allowed us to understand several features of stem cells in their natural intact environment (Doupé et al., 2012; Mascré et al., 2012; Simons and Clevers, 2011; Snippert et al., 2010; Van Keymeulen et al., 2011). The results obtained by analyzing the clones formed by stem cells at different time points after induction challenged the text-book view on how stem cells function *in vivo*. The long-assumed asymmetric way of cell division, in which a stem cell divides to generate an identical daughter and a differentiated cell (or a progenitor with limited differentiation or proliferative potential), was replaced by a neutral drift model in which stem cells prefer symmetric division to generate either two stem cells or two committed progenitors (Simons and Clevers, 2011). In this way, homeostasis (and the number of stem cells per organ/tissue) is maintained at the level of the niche, although clones expand or are eliminated while competing for space and/or niche resources.

In most vertebrate organs in which symmetric divisions are the rule among stem cells, homeostasis consists in the life-long maintenance of the proper number of differentiated cells, replacing lost cells by new ones. The fish retina constitutes a different system, in which homeostasis involves organ growth by the addition of new cells: older adult retinae have more cells than younger adult retinae. Our data show that RSCs undergo preferentially (but not only) asymmetric cell divisions. It is important to stress that our statements about asymmetric divisions focus on the cell types that are generated after mitoses, and not on the cellular components that might be involved in making the decision. Clones that are generated during late embryogenesis are still present and growing actively several months after, showing that they do not displace each other but rather extend parallel to the growth axis. This indicates that stemness among medaka RSCs is a very stable decision that guarantees maintaining clonal progression throughout life.

Notably, RSCs still prefer asymmetric divisions during response to injuries in the transient amplifying domain. This is in agreement with the absolute penetrance of RSC multipotency: RSCs do not choose between different options but rather do the very same thing irrespective of environmental conditions. Plastic responses to injuries rely on the progenitors rather than on stem cells, as evidenced by the transient nature of expansion or reduction of the clone's width that we observed. Along the same line, it was recently reported that both in the zebrafish and the *Xenopus* retina, the response to nutritional changes is mediated by RPCs and not by RSCs (Love et al., 2014). Progenitors have shorter cell cycles and therefore generate lots of cells in reduced periods of time to immediately respond to sudden cell loss. Allocating the task of a transient regenerative response to RPCs seems a superior solution to compromising RSCs, the actions of which will have a permanent impact and will be maintained during the entire life of the fish. However, we have not addressed the response of RSCs to ablations in the RSCs domain, but by definition, if they do regenerate the same cell type it can only be through symmetrical divisions. In the absence of this experiment, we cannot comment on the divisional preference of RSCs during regeneration of their own domain.

The results that we report here regarding the life-long activity of RSCs in the mature retina are in agreement with our previous results using transplantation experiments at blastula stages. As the induction of recombination at mature stages of the retina generates *i*ArCoSs that resemble the ArCoS obtained via transplantation, which reflect the behavior for RSCs at earlier stages, our results suggest that the RSC pool is established and fixed from the early retina development onwards. Whether this pool of stem cells is actively separated from the RPCs involved during the first wave of differentiation during retinogenesis or whether it is just the remains of cells that were not reached by the differentiation wave is still to be demonstrated.

Our data also show that the preference for predominant asymmetric divisions is maintained by RSCs throughout life. If there were a shift to symmetric divisions at some point during late adulthood, we would expect to observe the loss of straight *i*ArCoS and the appearance of a more stochastic scenario, with *i*ArCoSs disappearing or overexpanding. Even in the oldest fish analyzed, the shape of ArCoS and *i*ArCoS is maintained throughout life. The reliance on preferential asymmetric cell division constitutes a distinct feature of the adult CMZ, highlighting the behavioral spectrum of vertebrate post-embryonic stem cells and raising questions about the niche factors and molecular machinery that underpin its regulation.

## MATERIALS AND METHODS

### Fish stocks and generation of transgenic fish lines

Medaka (*Orizias latipes*) stocks were maintained as previously described (Rembold et al., 2006a). Transgenic lines were generated using a plasmid containing I-*Sce*I sites (pBS/I-*Sce*I) (Rembold et al., 2006a; Thermes et al., 2002). Fish were maintained in a fish facility built according to the local animal welfare standards (Tierschutzgesetz §11, Abs. 1, Nr. 1), and animal experiments were performed in accordance with European Union animal welfare guidelines. The facility is under the supervision of the local representative of the animal welfare agency.

#### Gaudí*^HspCre.A^*

The 1.7 kb zebrafish *Hsp70* promoter was cloned in a pBS/I-*Sce*I-containing a nuclear-localized, codon optimized CRE recombinase. The plasmid pBS/I-*Sce*I/*Hsp70*::Cre-NLS contains the insertional reporter *cmlc2*::EGFP.

#### Gaudí*^Ubiq.iCre^*

The 3.5 kb zebrafish *ubiquitin* promoter (Mosimann et al., 2011) (Addgene plasmid 27320) was cloned in a pBS/I-*Sce*I containing a tamoxifen-inducible Cre recombinase (from pIndu Perfect). The plasmid pBS/I-*Sce*I/*ubiquitin*::^ERT2^Cre contains the insertional reporter *cmlc2*::ECFP.

#### Gaudí^*RSG*^

The 3.5 kb zebrafish *ubiquitin* promoter replaced *Hsp70* promoter in Addgene plasmid 24334 (Hesselson et al., 2009). The plasmid pBS/I-SceI/*ubiquitin*::LoxP-DSRed-LoxP-H_2_B-EGFP contains a *cry*::ECFP as an insertional reporter. Gaudí^*RSG*^ transgenic fish have been successfully recombined over five generations.

#### Gaudí^*BBW2.1*^

The 3.5 kb zebrafish *ubiquitin* promoter (Mosimann et al., 2011) was subcloned in a pBS/I-*Sce*I containing an inverted BBW2.1 cassette (Livet et al., 2007), upstream of the Cerulean FP. Gaudí^*BBW2.1*^ transgenic fish have been successfully recombined for over six generations.

#### Gaudí*^LxBBW^*

A fragment contaning *ubiquitin*::LoxP-DSRed-LoxP from Gaudí^*RSG*^ was subcloned in a pBS/I-*Sce*I containing a BBW2.1 cassette, upstream of the Cerulean FP. Gaudí^LxBBW^ transgenic fish have been successfully recombined for over three generations.

#### LoxP^*OUT*^

Gaudí^*RSG*^ were outcrossed to Gaudí^*HspCre.A*^ and fish were heat-shocked and grown to adulthood. A female Gaudí^*RSG*^*,* Gaudí^*HspCre.A*^ produced embryos that were entirely green, irrespective of the *Hsp70*::Cre-NLS insertion and heat-shock treatment, indicating that recombination of the LoxP cassette happened in the germline of the mother and transmitted to the progeny. LoxP^*OUT*^ fish have been successfully maintained for over four generations.

### Injuries in the retina

Animals at 3 weeks post-hatching were anesthetized in 0.5× Tricaine (A5040, Sigma-Aldrich). Under microscopic visualization, the right retina was stabbed in the CMZ region in the dorsal and the two lateral quadrants with a glass needle (0.1 mm diameter). Only the tip of the needle was inserted to avoid injuring cells behind the CMZ. After treatment, the fish were returned to their tanks to recover. Left retinae were used as controls.

### Inducible expression of Cre recombinase

To induce CRE recombinase expression in Gaudí^*HspCre.A*^, juveniles were kept at 25°C for 3 h and transferred to ERM at 42°C. Gaudí^*Ubiq.iCre*^ juveniles were treated with a 2.5 μM tamoxifen (T5648 Sigma) solution for 3-24 h and washed afterwards with fish water. To favor sparse recombination of the Gaudí*^RSG^* line in the CMZ, we followed the same procedure using ERM at 36°C (for Gaudí^*HspCre.A*^) and 30-60 min of 2.5 μM tamoxifen (for Gaudí^*Ubiq.iCre*)^.

### Transplantation experiments

Transplantations were carried out as previously described (Ho and Kane, 1990; Rembold et al., 2006b). Ten to 15 Wimbledon^+/−^ blastula cells were transplanted into wild-type blastulae. Transplanted embryos were kept in 1× ERM supplemented with antibiotics (penicillin-streptomycin solution from Sigma, P0781, 1/200) and selected for EGFP^+^ cells.

### Antibodies and staining

Primary antibodies used in this study were rabbit anti-GFP (Invitrogen, A-11122, 1/750), chicken anti-GFP (Invitrogen, A-10262, 1/1000), rabbit α-DsRed (Clontech, 632496, 1/750), mouse anti-BrdU/IdU (Becton Dickinson, 347580, 1/25), rabbit anti-PKCα (Santa Cruz, sc-208 1/400), mouse anti-PCNA (Santa Cruz, sc-56, 1/400), rabbit anti-phospho-histone 3 (Upstate, 06-570, 1/500), mouse anti-HuC (Invitrogen, A-21271, 1/200), mouse anti-GS (BD Biosciences, 610517, 1/500), mouse anti-parvalbumin (Chemicon, MAB1572, 1/400) (Inoue and Wittbrodt, 2011). Secondary antibodies were Alexa488 anti-rabbit and Alexa546 anti-mouse (Invitrogen, A-11034 and A-11030 respectively, 1/400), and DyLight488 anti-chicken, DyLight549 anti-rabbit and Cy5 anti-mouse (Jackson, 703-485-155, 112-505-144 and 715-175-151, respectively, 1/400).

### Imaging

Samples were imaged using an Olympus MVX10 binocular coupled to a Leica DFC500 camera (living juveniles, entire retina), a Nikon AZ100 coupled to a Nikon C1 (entire retinae), and a Leica TCS SP5.

## Supplementary Material

Supplementary Material

## References

[DEV109892C1] Allison, W. T., Barthel, L. K., Skebo, K. M., Takechi, M., Kawamura, S. and Raymond, P. A. (2010). Ontogeny of cone photoreceptor mosaics in zebrafish. *J. Comp. Neurol.* 518, 4182-4195 10.1002/cne.22447 20878782 PMC3376642

[DEV109892C2] Amato, M. A., Arnault, E. and Perron, M. (2004). Retinal stem cells in vertebrates: parallels and divergences. *Int. J. Dev. Biol.* 48, 993-1001 10.1387/ijdb.041879ma 15558490

[DEV109892C3] Blanpain, C. and Fuchs, E. (2009). Epidermal homeostasis: a balancing act of stem cells in the skin. *Nat. Rev. Mol. Cell Biol.* 10, 207-217 10.1038/nrm2636 19209183 PMC2760218

[DEV109892C4] Bonaguidi, M. A., Wheeler, M. A., Shapiro, J. S., Stadel, R. P., Sun, G. J., Ming, G.-L. and Song, H. (2011). In vivo clonal analysis reveals self-renewing and multipotent adult neural stem cell characteristics. *Cell* 145, 1142-1155 10.1016/j.cell.2011.05.024 21664664 PMC3124562

[DEV109892C5] Brock, A., Chang, H. and Huang, S. (2009). Non-genetic heterogeneity--a mutation-independent driving force for the somatic evolution of tumours. *Nat. Rev. Genet.* 10, 336-342 10.1038/nrg2556 19337290

[DEV109892C6] Centanin, L. and Wittbrodt, J. (2014). Retinal neurogenesis. *Development* 141, 241-244 10.1242/dev.083642 24381194

[DEV109892C7] Centanin, L., Hoeckendorf, B. and Wittbrodt, J. (2011). Fate restriction and multipotency in retinal stem cells. *Cell Stem Cell* 9, 553-562 10.1016/j.stem.2011.11.004 22136930

[DEV109892C8] Costa, M. R., Ortega, F., Brill, M. S., Beckervordersandforth, R., Petrone, C., Schroeder, T., Götz, M. and Berninger, B. (2011). Continuous live imaging of adult neural stem cell division and lineage progression in vitro. *Development* 138, 1057-1068 10.1242/dev.061663 21343361

[DEV109892C9] Doupé, D. P., Alcolea, M. P., Roshan, A., Zhang, G., Klein, A. M., Simons, B. D. and Jones, P. H. (2012). A single progenitor population switches behavior to maintain and repair esophageal epithelium. *Science* 337, 1091-1093 10.1126/science.1218835 22821983 PMC3527005

[DEV109892C10] Encinas, J. M., Michurina, T. V., Peunova, N., Park, J.-H., Tordo, J., Peterson, D. A., Fishell, G., Koulakov, A. and Enikolopov, G. (2011). Division-coupled astrocytic differentiation and age-related depletion of neural stem cells in the adult hippocampus. *Cell Stem Cell* 8, 566-579 10.1016/j.stem.2011.03.010 21549330 PMC3286186

[DEV109892C11] Fekete, D. M., Perez-Miguelsanz, J., Ryder, E. F. and Cepko, C. L. (1994). Clonal analysis in the chicken retina reveals tangential dispersion of clonally related cells. *Dev. Biol.* 166, 666-682 10.1006/dbio.1994.1346 7813785

[DEV109892C12] Fox, D., Morris, L., Nystul, T. and Spradling, A. (2008). *Lineage Analysis of Stem Cells*. Cambridge, MA: Harvard Stem Cell Institute. 20614627

[DEV109892C13] Gomes, F. L. A. F., Zhang, G., Carbonell, F., Correa, J. A., Harris, W. A., Simons, B. D. and Cayouette, M. (2011). Reconstruction of rat retinal progenitor cell lineages in vitro reveals a surprising degree of stochasticity in cell fate decisions. *Development* 138, 227-235 10.1242/dev.059683 21148186 PMC3005599

[DEV109892C14] Graf, T. and Stadtfeld, M. (2008). Heterogeneity of embryonic and adult stem cells. *Cell Stem Cell* 3, 480-483 10.1016/j.stem.2008.10.007 18983963

[DEV109892C15] Gupta, V. and Poss, K. D. (2012). Clonally dominant cardiomyocytes direct heart morphogenesis. *Nature* 484, 479-484 10.1038/nature11045 22538609 PMC3340018

[DEV109892C16] Hans, S., Kaslin, J., Freudenreich, D. and Brand, M. (2009). Temporally-controlled site-specific recombination in zebrafish. *PLoS ONE* 4, e4640 10.1371/journal.pone.0004640 19247481 PMC2645673

[DEV109892C17] He, J., Zhang, G., Almeida, A. D., Cayouette, M., Simons, B. D. and Harris, W. A. (2012). How variable clones build an invariant retina. *Neuron* 75, 786-798 10.1016/j.neuron.2012.06.033 22958820 PMC3485567

[DEV109892C18] Hesselson, D., Anderson, R. M., Beinat, M. and Stainier, D. Y. R. (2009). Distinct populations of quiescent and proliferative pancreatic beta-cells identified by HOTcre mediated labeling. *Proc. Natl. Acad. Sci. USA* 106, 14896-14901 10.1073/pnas.0906348106 19706417 PMC2736433

[DEV109892C19] Ho, R. K. and Kane, D. A. (1990). Cell-autonomous action of zebrafish spt-1 mutation in specific mesodermal precursors. *Nature* 348, 728-730 10.1038/348728a0 2259382

[DEV109892C20] Holt, C. E., Bertsch, T. W., Ellis, H. M. and Harris, W. A. (1988). Cellular determination in the Xenopus retina is independent of lineage and birth date. *Neuron* 1, 15-26 10.1016/0896-6273(88)90205-X 3272153

[DEV109892C21] Inoue, D. and Wittbrodt, J. (2011). One for all--a highly efficient and versatile method for fluorescent immunostaining in fish embryos. *PLoS ONE* 6, e19713 10.1371/journal.pone.0019713 21603650 PMC3094454

[DEV109892C22] Johns, P. R. (1977). Growth of the adult goldfish eye. III. Source of the new retinal cells. *J. Comp. Neurol.* 176, 343-357 10.1002/cne.901760304 915042

[DEV109892C23] Klein, A. M., Nakagawa, T., Ichikawa, R., Yoshida, S. and Simons, B. D. (2010). Mouse germ line stem cells undergo rapid and stochastic turnover. *Cell Stem Cell* 7, 214-224 10.1016/j.stem.2010.05.017 20682447

[DEV109892C24] Knopf, F., Hammond, C., Chekuru, A., Kurth, T., Hans, S., Weber, C. W., Mahatma, G., Fisher, S., Brand, M. and Schulte-Merker, S. et al. (2011). Bone regenerates via dedifferentiation of osteoblasts in the zebrafish fin. *Dev. Cell* 20, 713-724 10.1016/j.devcel.2011.04.014 21571227

[DEV109892C25] Li, Y., Lu, H., Cheng, P.-L., Ge, S., Xu, H., Shi, S.-H. and Dan, Y. (2012). Clonally related visual cortical neurons show similar stimulus feature selectivity. *Nature* 486, 118-121. 22678292 10.1038/nature11110 PMC3375857

[DEV109892C26] Livet, J., Weissman, T. A., Kang, H., Draft, R. W., Lu, J., Bennis, R. A., Sanes, J. R. and Lichtman, J. W. (2007). Transgenic strategies for combinatorial expression of fluorescent proteins in the nervous system. *Nature* 450, 56-62 10.1038/nature06293 17972876

[DEV109892C27] Loulier, K., Barry, R., Mahou, P., Le Franc, Y., Supatto, W., Matho, K. S., Ieng, S., Fouquet, S., Dupin, E. and Benosman, R. et al. (2014). Multiplex cell and lineage tracking with combinatorial labels. *Neuron* 81, 505-520 10.1016/j.neuron.2013.12.016 24507188

[DEV109892C28] Love, N. K., Keshavan, N., Lewis, R., Harris, W. A. and Agathocleous, M. (2014). A nutrient-sensitive restriction point is active during retinal progenitor cell differentiation. *Development* 141, 697-706 10.1242/dev.103978 24449845 PMC3899821

[DEV109892C29] Margolis, J. and Spradling, A. (1995). Identification and behavior of epithelial stem cells in the Drosophila ovary. *Development* 121, 3797-3807. 8582289 10.1242/dev.121.11.3797

[DEV109892C30] Mascré, G., Dekoninck, S., Drogat, B., Youssef, K. K., Brohée, S., Sotiropoulou, P. A., Simons, B. D. and Blanpain, C. (2012). Distinct contribution of stem and progenitor cells to epidermal maintenance. *Nature* 489, 257-262 10.1038/nature11393 22940863

[DEV109892C31] Mizutani, K.-I., Yoon, K., Dang, L., Tokunaga, A. and Gaiano, N. (2007). Differential Notch signalling distinguishes neural stem cells from intermediate progenitors. *Nature* 449, 351-355 10.1038/nature06090 17721509

[DEV109892C32] Mosimann, C., Kaufman, C. K., Li, P., Pugach, E. K., Tamplin, O. J. and Zon, L. I. (2011). Ubiquitous transgene expression and Cre-based recombination driven by the ubiquitin promoter in zebrafish. *Development* 138, 169-177 10.1242/dev.059345 21138979 PMC2998170

[DEV109892C33] Nakamura, S., Kobayashi, K., Nishimura, T., Higashijima, S.-i. and Tanaka, M. (2010). Identification of germline stem cells in the ovary of the teleost medaka. *Science* 328, 1561-1563 10.1126/science.1185473 20488987

[DEV109892C34] Ohlstein, B. and Spradling, A. (2006). The adult Drosophila posterior midgut is maintained by pluripotent stem cells. *Nature* 439, 470-474 10.1038/nature04333 16340960

[DEV109892C35] Pan, Y. A., Freundlich, T., Weissman, T. A., Schoppik, D., Wang, X. C., Zimmerman, S., Ciruna, B., Sanes, J. R., Lichtman, J. W. and Schier, A. F. (2013). Zebrabow: multispectral cell labeling for cell tracing and lineage analysis in zebrafish. *Development* 140, 2835-2846 10.1242/dev.094631 23757414 PMC3678346

[DEV109892C36] Price, J., Turner, D. and Cepko, C. (1987). Lineage analysis in the vertebrate nervous system by retrovirus-mediated gene transfer. *Proc. Natl. Acad. Sci. USA* 84, 156-160 10.1073/pnas.84.1.156 3099292 PMC304161

[DEV109892C37] Reh, T. A. and Levine, E. M. (1998). Multipotential stem cells and progenitors in the vertebrate retina. *J. Neurobiol.* 36, 206-220 10.1002/(SICI)1097-4695(199808)36:2<206::AID-NEU8>3.0.CO;2-5 9712305

[DEV109892C38] Rembold, M., Lahiri, K., Foulkes, N. S. and Wittbrodt, J. (2006a). Transgenesis in fish: efficient selection of transgenic fish by co-injection with a fluorescent reporter construct. *Nat. Protoc.* 1, 1133-1139 10.1038/nprot.2006.165 17406394

[DEV109892C39] Rembold, M., Loosli, F., Adams, R. J. and Wittbrodt, J. (2006b). Individual cell migration serves as the driving force for optic vesicle evagination. *Science* 313, 1130-1134 10.1126/science.1127144 16931763

[DEV109892C40] Rinkevich, Y., Lindau, P., Ueno, H., Longaker, M. T. and Weissman, I. L. (2011). Germ-layer and lineage-restricted stem/progenitors regenerate the mouse digit tip. *Nature* 476, 409-413 10.1038/nature10346 21866153 PMC3812235

[DEV109892C41] Rothenaigner, I., Krecsmarik, M., Hayes, J. A., Bahn, B., Lepier, A., Fortin, G., Götz, M., Jagasia, R. and Bally-Cuif, L. (2011). Clonal analysis by distinct viral vectors identifies bonafide neural stem cells in the adult zebrafish telencephalon and characterizes their division properties and fate. *Development* 138, 1459-1469 10.1242/dev.058156 21367818

[DEV109892C42] Simons, B. D. and Clevers, H. (2011). Strategies for homeostatic stem cell self-renewal in adult tissues. *Cell* 145, 851-862 10.1016/j.cell.2011.05.033 21663791

[DEV109892C43] Singh, S. P., Holdway, J. E. and Poss, K. D. (2012). Regeneration of amputated zebrafish fin rays from de novo osteoblasts. *Dev. Cell* 22, 879-886 10.1016/j.devcel.2012.03.006 22516203 PMC3341140

[DEV109892C44] Snippert, H. J., van der Flier, L. G., Sato, T., van Es, J. H., van den Born, M., Kroon-Veenboer, C., Barker, N., Klein, A. M., van Rheenen, J. and Simons, B. D. et al. (2010). Intestinal crypt homeostasis results from neutral competition between symmetrically dividing Lgr5 stem cells. *Cell* 143, 134-144 10.1016/j.cell.2010.09.016 20887898

[DEV109892C45] Thermes, V., Grabher, C., Ristoratore, F., Bourrat, F., Choulika, A., Wittbrodt, J. and Joly, J.-S. (2002). I-SceI meganuclease mediates highly efficient transgenesis in fish. *Mech. Dev.* 118, 91-98 10.1016/S0925-4773(02)00218-6 12351173

[DEV109892C46] Tu, S. and Johnson, S. L. (2011). Fate restriction in the growing and regenerating zebrafish fin. *Dev. Cell* 20, 725-732 10.1016/j.devcel.2011.04.013 21571228 PMC3096007

[DEV109892C47] Van Keymeulen, A., Rocha, A. S., Ousset, M., Beck, B., Bouvencourt, G., Rock, J., Sharma, N., Dekoninck, S. and Blanpain, C. (2011). Distinct stem cells contribute to mammary gland development and maintenance. *Nature* 479, 189-193 10.1038/nature10573 21983963

[DEV109892C48] Wetts, R. and Fraser, S. E. (1988). Multipotent precursors can give rise to all major cell types of the frog retina. *Science* 239, 1142-1145 10.1126/science.2449732 2449732

[DEV109892C49] Wetts, R., Serbedzija, G. N. and Fraser, S. E. (1989). Cell lineage analysis reveals multipotent precursors in the ciliary margin of the frog retina. *Dev. Biol.* 136, 254-263 10.1016/0012-1606(89)90146-2 2478403

[DEV109892C50] Wong, L. L. and Rapaport, D. H. (2009). Defining retinal progenitor cell competence in Xenopus laevis by clonal analysis. *Development* 136, 1707-1715 10.1242/dev.027607 19395642 PMC2673759

[DEV109892C51] Yu, Y.-C., He, S., Chen, S., Fu, Y., Brown, K. N., Yao, X.-H., Ma, J., Gao, K. P., Sosinsky, G. E. and Huang, K. et al. (2012). Preferential electrical coupling regulates neocortical lineage-dependent microcircuit assembly. *Nature* 486, 113-117. 22678291 10.1038/nature10958 PMC3599787

[DEV109892C52] Zhu, X., Hill, R. A., Dietrich, D., Komitova, M., Suzuki, R. and Nishiyama, A. (2011). Age-dependent fate and lineage restriction of single NG2 cells. *Development* 138, 745-753 10.1242/dev.047951 21266410 PMC3026417

