# Supplementary material for: Exclusive multipotency and preferential asymmetric divisions in post-embryonic neural stem cells of the fish retina
Source: Development. 2014 Sep;141(18):3472–82. doi: 10.1242/dev.109892 (PMC4197724; doi:10.1242/dev.109892)
Supplement: Supplementary Material [file supp_141_18_3472__index.html]

Exclusive multipotency and preferential asymmetric divisions in post-embryonic neural stem cells of the fish retina — Supplementary Material 

# Exclusive multipotency and preferential asymmetric divisions in post-embryonic neural stem cells of the fish retina

## DEV109892 Supplementary Material

**Files in this Data Supplement:**

- **Supplementary Material**
