# Supplementary material for: Exclusive multipotency and preferential asymmetric divisions in post-embryonic neural stem cells of the fish retina
Source: Development. 2014 Sep;141(18):3472–82. doi: 10.1242/dev.109892 (PMC4197724; doi:10.1242/dev.109892)
Supplement: Supplementary Material [file supp_141.18.3472_DEV109892.pdf]

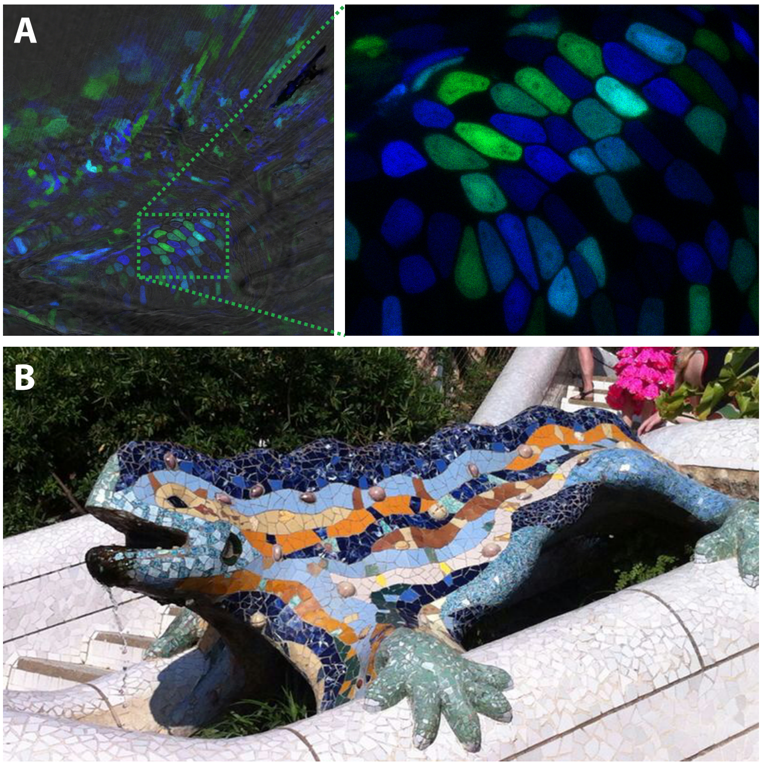

Centanin et al. Fig. S1. Generation of multicolor mosaics in medaka. (**A,B**) Induced CRE-NLS recombination in a medaka juvenile expressing ubiquitously a Brainbow cassette generates a colorful mosaic pattern (A) that resembles the famous statues of Parc Güel by Antoni Gaudí (B) - photo courtesy of Alicia Perez-Saturnino.

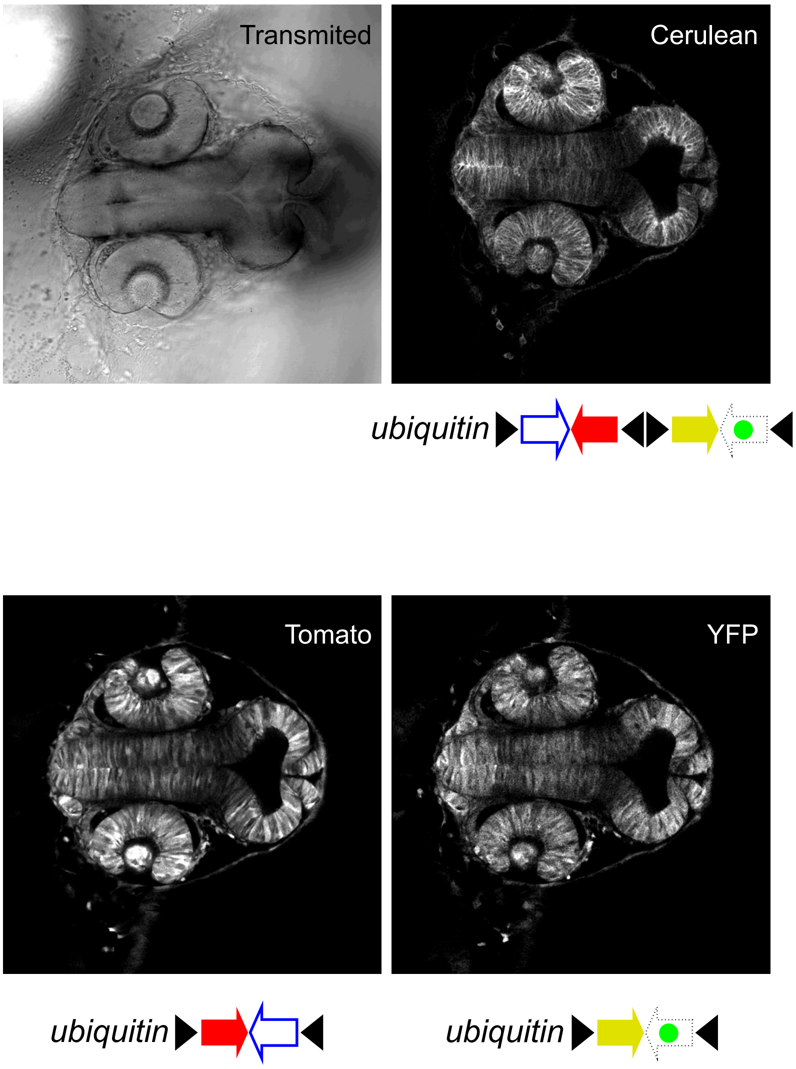

Centanin et al. Fig. S2. Heat-shock mediated recombination in medaka embryos. Induction of a Cre-NLS recombinase by a heat-shock treatment results in LoxP mediated recombination of Gaudí$^{BBW2.1}$ medaka embryos. Besides the default Cerulean fluorescent protein (top right), recombined embryos show also Tomato (bottom left) and eYFP (bottom right) expression in different embryonic tissues.

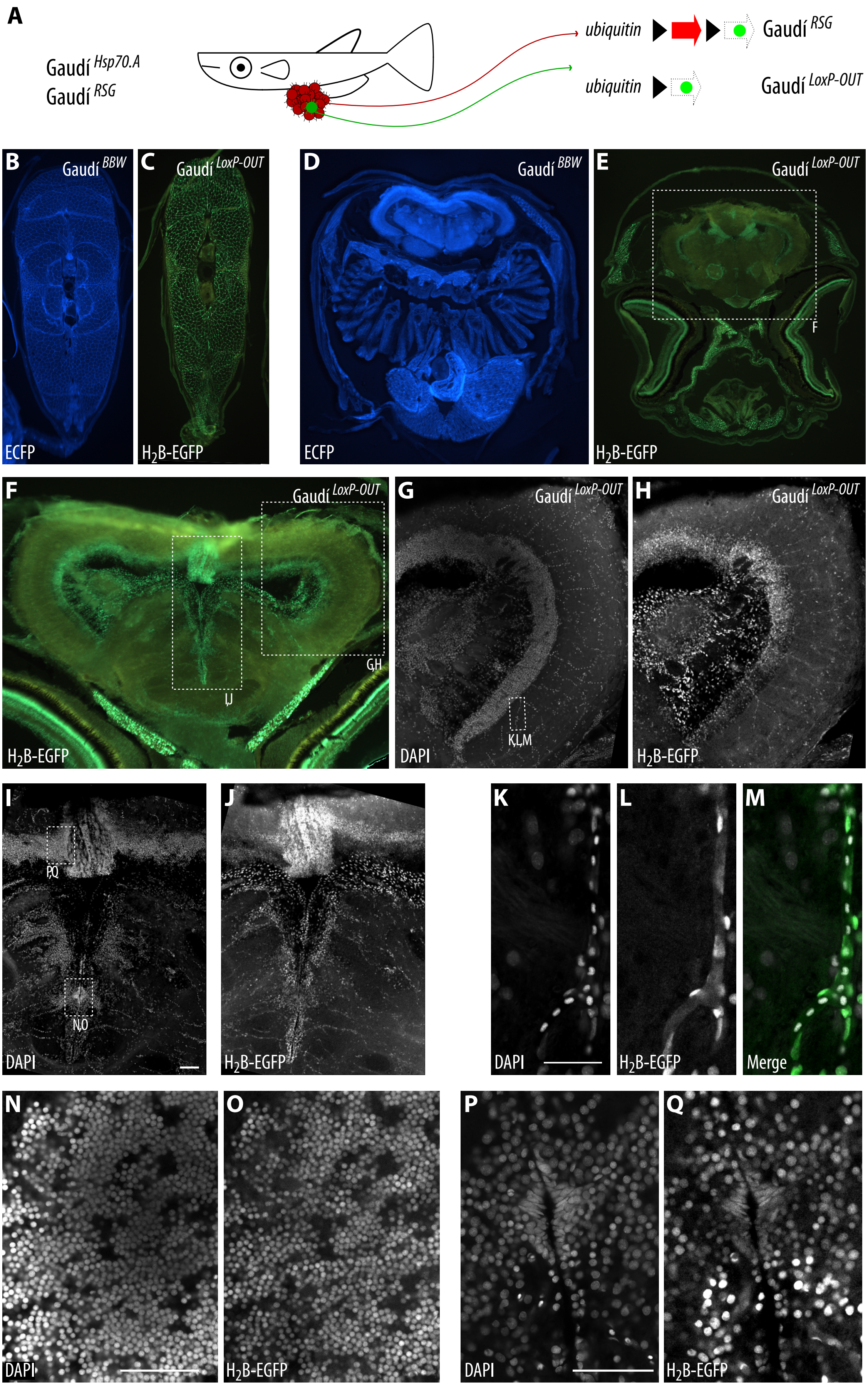

Centanin et al., Fig. S3. Gaudí reporter lines are ubiquitously expressed also in adult tissues. **(A)** Inducing recombination in Gaudí^{RSG} can trigger labeling in germ cells, and therefore produce in the next generation fish bearing the transgene already edited, Gaudí$^{LoxP\text{-}OUT}$ (*ubiquitin*::LoxP-H2B-EGFP). Gaudí$^{LoxP\text{-}OUT}$ fish constitute an ideal tool to address ubiquitous expression, since nuclear-tagged fluorescent proteins are unambiguously assigned to DAPI signals. **(B-E)** Sections trough the somites and brain show ubiquitous expression in Gaudí$^{BBW2.1}$ and Gaudí$^{LoxP\text{-}OUT}$.**(F-Q)** Confocal analysis reveals that Gaudí$^{LoxP\text{-}OUT}$ express H2B-EGFP ubiquitously, as shown by counterstaining with DAPI, in every cell type with the exception of the blood cells

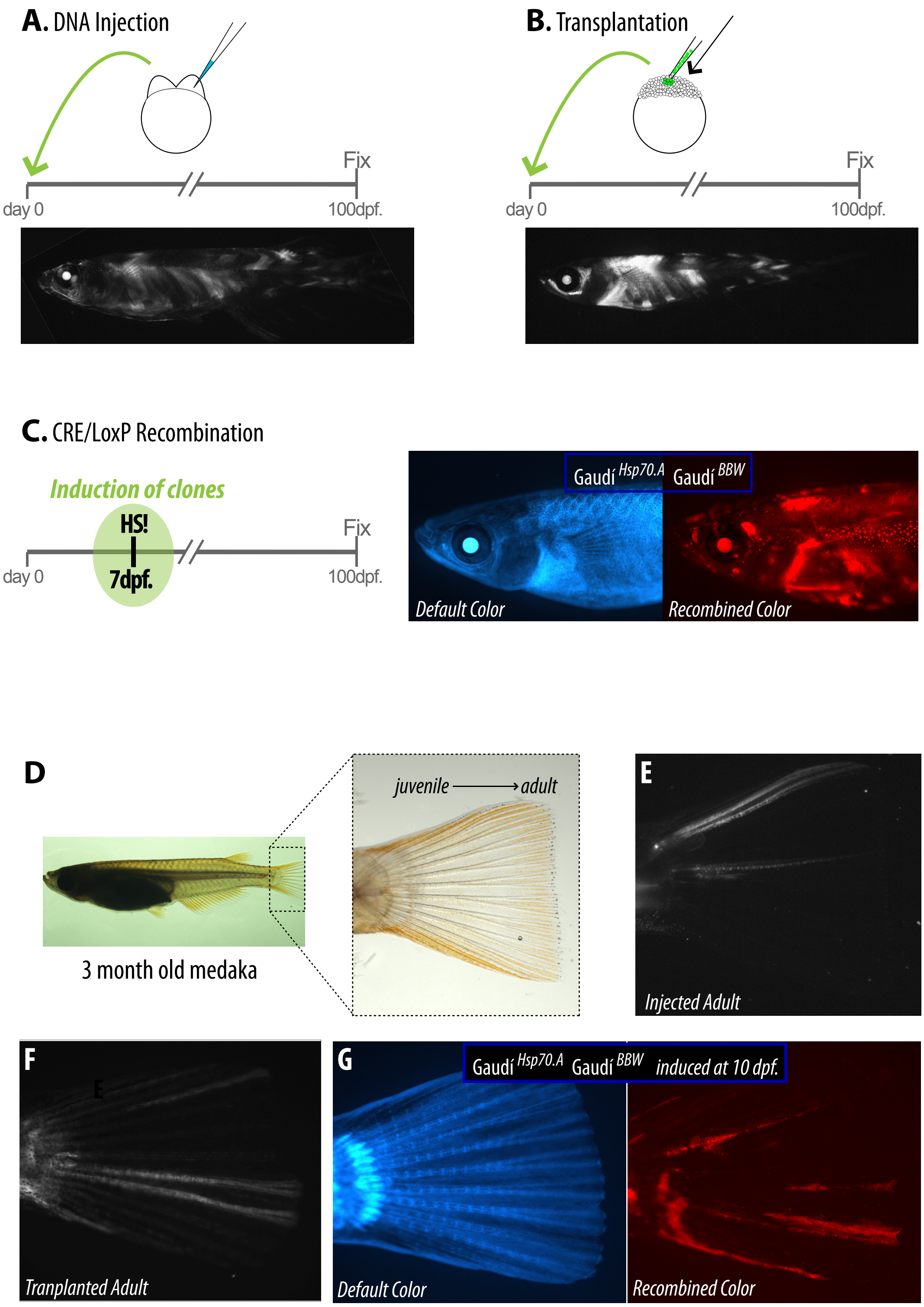

Centanin et al., Fig. S4. Long term lineage analysis using the Gaudí toolkit. (**A, B**) Labeling early embryonic cell by DNA injection (A) or by transplanting cells from a labelled transgenic donor blastula (B) generates adult fish in which the labelled clones represent the lineage of the stem cells labelled during embryogenesis. (**C**) Juvenile induction of Gaudí fish allows lineage of post-embryonic stem cells until adulthood. (**D-G**) The fish tail fin growth by addition of new cells from post-embryonic stem cells, a stereotyped spatio-temporal process (D). Clones generated by DNA injection (E) or blastula transplantation (F) reveal the activity of stem cells labelled during early embryogenesis. The Gaudí toolkit allows following instead the lineage of post-embryonic stem

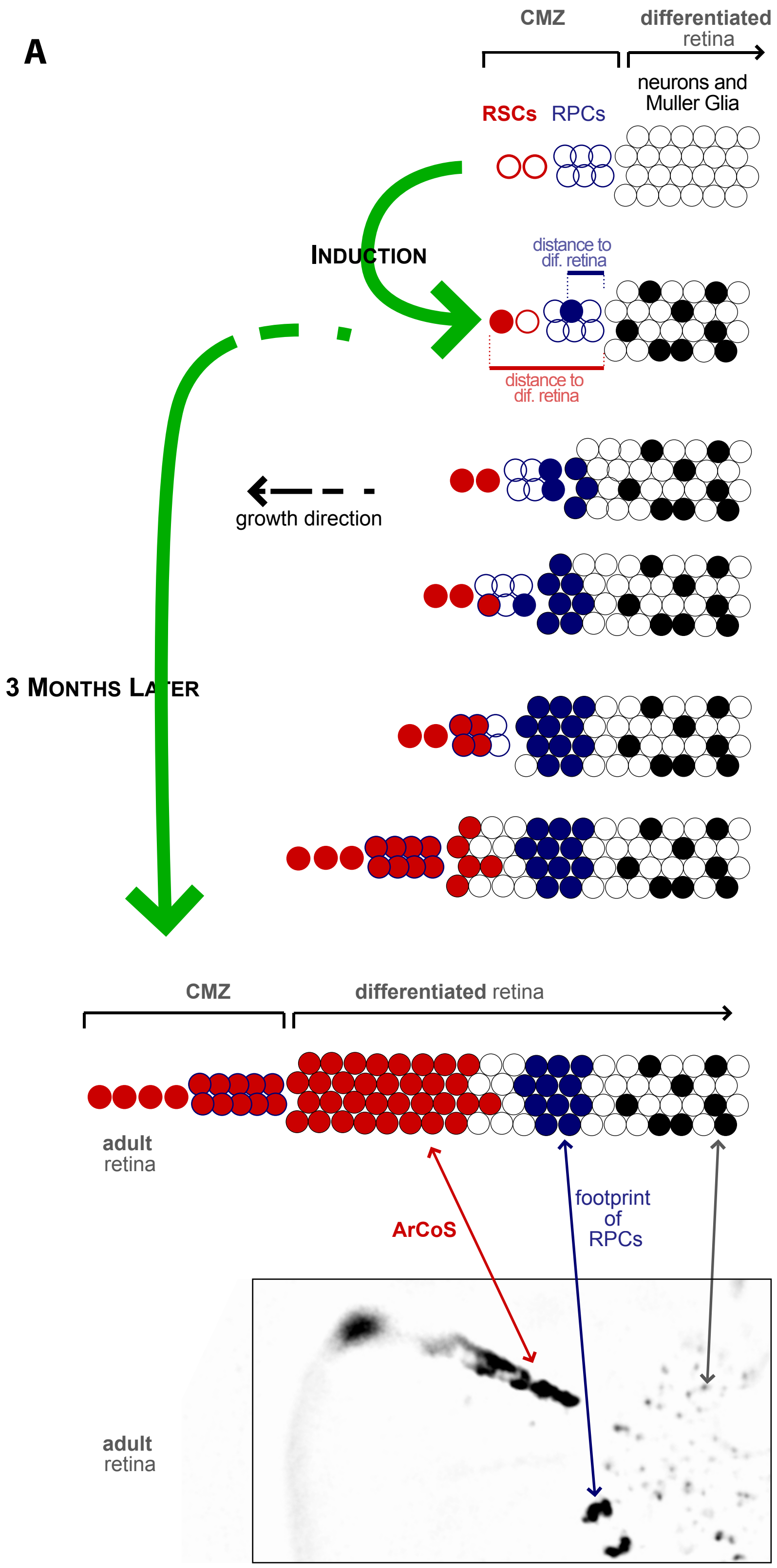

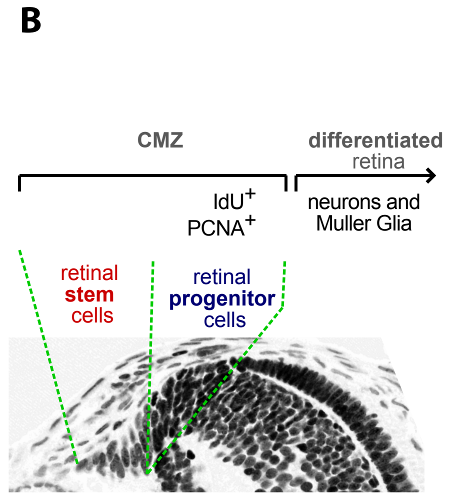

Centanin et al. Fig. S5. Differential location for RSCs and RPCs within the CMZ. (**A**) After CRE-mediated recombination, different cells within the CMZ are labeled. RPCs generate smaller clones due to their transient nature, and RSCs are long-lived and generate ArCoSs. The footprints of RPCs map closer to the embryonic retina than the first differentiated cells in an ArCoS. (B) These differences in the localization of clones reflect the more central location of RPCs in the CMZ and the more peripheral position of genuine RSCs.

Captions for the Movies
.

Movie 1. Clone produced by a RPC, chase of 7 days. The entire progeny of the founder RPC is differentiated and locates to the layered neural retina. The clone does not have any contact with the CMZ.

Movie M2. Clone produced by a RSC, chase of 7 days. The progeny of the founder RSC has colonized the transient amplifying domain and some cells of the clone are already integrated in the layered neural retina. The clone continues within the CMZ.

Movie M3. Clone produced by a RSC, chase of 20 days. The progeny of the founder RSCs has already formed an *i*ArCoS. Note that the clone is continuous with the CMZ, and that the *i*ArCoS expands through the 3 layers of the differentiated neural retina.

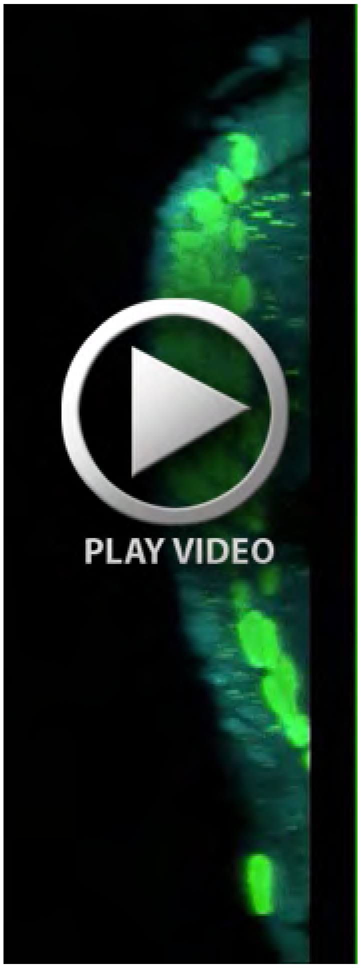

Movie 1.

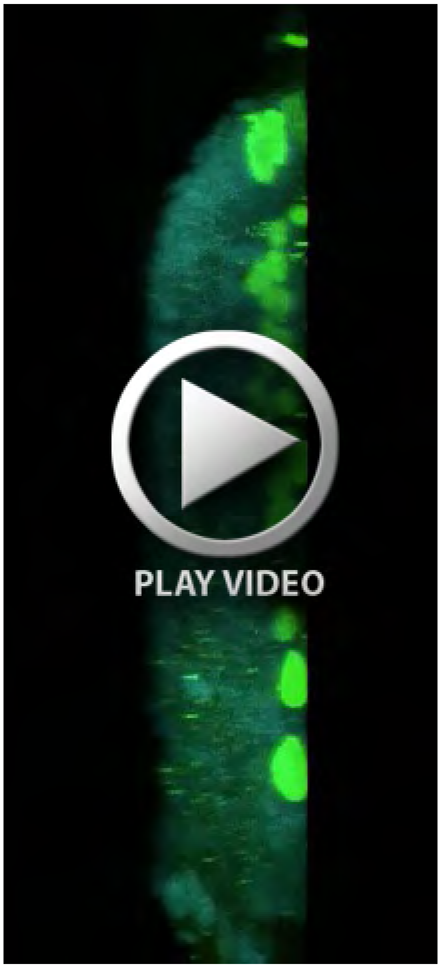

Movie 2.

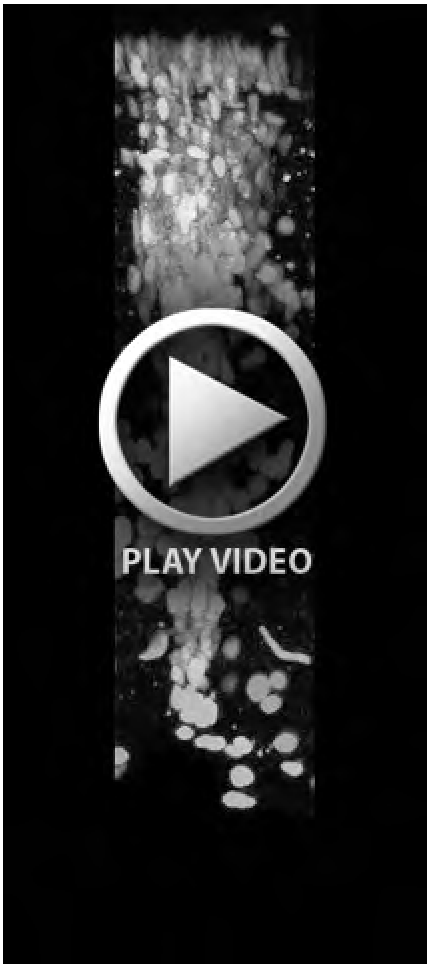

Movie 3.
